# The Treatment of Teeth Having Putresent Pulps

**Published:** 1898-03

**Authors:** F. P. Hamley

**Affiliations:** Hempstead, N. Y.


ARTICLE II.

### THE TREATMENT OF TEETH HAVING PUTRESENT PULPS.

BY DR. F. P. HAMLEY, HEMPSTEAD, N. Y.

Read before Second District Dental Society, Newburg, N. Y., Oc. 11, '97.

Treatment may be said to be experimental. Knowledge obtained through experimentation is the most valuable, and for the treatment of disease it is always best to prescribe that treatment which has been founded on experiment and practice—for "experience is the best teacher."

But in the treatment of diseased teeth, we never seem to have experience enough. Although we may flatter ourselves that we are capable of soothing any ill that a tooth may have, we find times when that experimental knowledge fails. Then we do not all have the same ideas about disease, or the treatment of disease, and we are treating teeth variously as the result of individual experiments.

The question then arises—Should we adhere to any fixed treatment in our efforts to save diseased teeth ? You can appreciate what I mean when you call to mind that severe case of toothache, upon which you have applied all the remedies in your cabinet with but little relief to your patient, and after many vain attempts to soothe the pain, you go to your supply of drugs and discover some medicine, or combination of medicines, which you have never tried before, and upon applying it, the sufferer is relieved.

Medicines do *not* then give the same results at all times, and for this reason the treatment of disease must necessarily be experimental But this experimenting must proceed with intelligence. The operator must possess some knowledge of diseased conditions, and also know the actions of the medicines he has at his command.

The young graduate finds his first patient suffering with toothache. He cannot determine from an examination what is the cause or the nature of the trouble. From the apparent suffering of his patient, he decides to "kill the nerve" as a means to stop the ache, so he proceeds to use arsenious acid, applying it, though unconsciously, to a nerve already congested, and inflamed. The patient—"Oh, where is he ?"—crazed with pain, and likely to suffer long after the pulp is dead. What is the cause of this prolonged suffering ? The irritation caused by the action of the arsenious acid has inflamed the inner membranes, found its way through the dental tubes, destroying the albumen, cutting off blood supply. But before it has caused such utter destruction, the tooth is filled and may keep quiet for months, perhaps years, or until the effects of the poison reaches the cementum; the tooth then becomes a foreign substance—as foreign to the delicate membrane as a piece of wood. Nature rebels. Pericementitis sets in, and it is an act of mercy to extract the tooth before greater trouble asserts itself.

Some mechanical or surgical means to extirpate a pulp is far better than the unlimited use of destroying agents. A tooth can certainly be treated and filled in less time, and with better results.

The treatment of devitalized teeth is wearisome indeed, and the length of their usefulness is limited. We have men who boldly assert that they never have trouble with a devitalized tooth, because their method is so thoroughly effectual. In fact there is nothing left to cause after trouble. How do they know there is nothing left? Is it that their sense of smell is so keen? Their touch is delicate? Their sight so perfect? Or by the number of times the cavity is washed? Certainly they must have some means of knowing just when the tooth is sufficiently treated, so that forever after it will be peaceful. It seems to me quite impossible to know this without a doubt. We cleanse the tooth to *our* satisfaction, but that does not necessarily mean that nature approves of it. Nature becomes offended at slight causes. We remove pulp in all stages of decomposition, and though we are careful to follow that method, which is thoroughly clean, we are nevertheless sometimes disappointed in our efforts.

The best therapeutic medicaments for the treatment of diseased or devitalized teeth, are those which meet the cenditions.

In a tooth where the pulp has already dissolved, becoming putrescent, we have a pathological condition which requires the greatest care. The medicaments must possess, first, the power of destroying the products of putrefaction; secondly, the power of destroying the agents of putrefaction; and thirdly, to have the power of coagulating or permeating the remains of the dental fibres.

Sulphurated hydrogen gas is the product of putrefaction, which is generated by the decomposition of dead pulp. Decompose the gas by using iodoform, also a deodorant—remove all dead matter and we are ready for the second pathological condition—the "agent of putrefaction"—wash thoroughly with oil cinnamon or eucalyptus, the former preferred, seal the tooth temporarily, leaving oil cinnamon in canals for a week or more.

The third pathological condition can be best treated by forcing into the remaining tooth some preserving fluid —a fluid which will incorporate with dead albumen. Chloride of zinc or creosote, or both in combination, has proven satisfactory and generally reliable. It readily permeates the dentinal tubuli and puts the remaining tooth structure in a state of health.

We have then three conditions to meet in the treatment of diseased teeth before filling them :

1. To destroy the products of putrefaction.

2. To destroy the agent of putrefaction.

3. To charge the tooth with an agent of preservation.

Chloride zinc being a coagulant, converts all the fluid matter remaining in the tubuli into a consistent state, and for that reason great care should be exercised in applying it at the right time.

A very extensive paper treating of this subject was read by Dr. A. W. Harlan at a dental meeting held in New York City. Dr. Harlan claims that the use of a coagulant at the first stage of treatment, closes up the mouths of the tubuli and prevents antiseptics going through the tooth, while Dr. Barrett, in the *Dental Advertiser*, claims that the very first requisite is coagulation, and that coagulation is the very thing that should be brought about.

Dr. Barrett then attacks the pathological conditions in this order :

1. "Coagulation of the dentinal fibre."

2. "An opportunity to melt down." (To dissolve, I presume, is meant.)

3. Careful sterilization.

3. Sealing of the tubuli.

He uses coagulants, while Dr. Harlan discards them.

My order of treating putrescent pulps may occasionally fail, but I have faith in it for the majority of cases which come under my care. First, open the pulp cavity well if it has not already been done, and before removing any of the putrescent matter, destroy the product of putrefaction which is the sulphuretted hydrogen gas, formed by the decomposed matter. For this I use iodoform carried into the tooth in powder form. Then destroy the agent of putrefaction by washing with antiseptics and sealing the cavity, having first charged it liberally with oil of cinnamon, or eucalyptus, the former preferred, letting it remain closed for several days.—*Items of Interest.*

